# Primary Extra-Nodal DLBCL of Glands: Our Experiences outside Guidelines of Treatment

**DOI:** 10.3390/healthcare9030286

**Published:** 2021-03-05

**Authors:** Antonello Sica, Mario Santagata, Caterina Sagnelli, Piero Rambaldi, Renato Franco, Massimiliano Creta, Paola Vitiello, Stefano Caccavale, Vincenzo Tammaro, Evangelista Sagnelli, Andrea Ronchi

**Affiliations:** 1 Department of Precision Medicine, University of Campania “Luigi Vanvitelli”, 80131 Naples, Italy; antonello.sica@fastwebnet.it (A.S.); pierfrancesco.rambaldi@unicampania.it (P.R.); 2 Multidisciplinary Department of Medical Surgery and Dental Specialties, University of Campania “Luigi Vanvitelli”, 80131 Naples, Italy; mario.santagata@unicampania.it; 3 Department of Mental Health and Public Medicine, University of Campania “Luigi Vanvitelli”, 80138 Naples, Italy; evangelista.sagnelli@unicampania.it; 4 Division of Pathology, Department of Mental Health and Preventive, University of Campania “Luigi Vanvitelli”, 80138 Naples, Italy; renato.franco@unicampania.it (R.F.); andrea.ronchi@unicampania.it (A.R.); 5 Department of Neurosciences, Reproductive Sciences and Odontostomatology, University of Naples “Federico II”, 80131 Naples, Italy; massimiliano.creta@unina.it; 6 Dermatology Unit, University of Campania “Luigi Vanvitelli”, 80138 Naples, Italy; paoladermosun@gmail.com (P.V.); stefano85med@gmail.com (S.C.); 7 Department of Advanced Biomedical Sciences, University of Naples Federico II, 80131 Naples, Italy; vincenzo.tammaro@unina.it

**Keywords:** primary extra-nodal lymphoma, thyroid lymphoma, parotid gland lymphoma, non-Hodgkin lymphoma

## Abstract

Lymphomas usually involve lymph nodes and other lymphoid tissues, but sometimes occur in non-lymphoid organs, called extra-nodal sites. Primary diffuse extra-lymph node large B-cell lymphoma (DLBCL) of the thyroid and parotid gland have been observed rarely. According to the most accredited guidelines, primary extra-nodal DLBCL of the parotid and thyroid glands should be treated with three cycles of R-CHOP followed by radiotherapy of the involved site (ISRT). Surgery alone is not enough to treat DLBCL. We describe two unusual cases of primary extra-nodal DLBCL in elderly patients treated exclusively with surgical resection, given the inability to apply chemotherapy. Both patients achieved clinical recovery, which was maintained after a follow-up of more than 18 months, despite not having performed the indicated chemotherapy protocol. The two cases presented here, and a few others reported in the literature, should be considered exceptions to the rule, and do not allow the conclusion that surgery alone might be sufficient for complete remission.

## 1. Introduction

Diffuse large B-cell lymphoma (DLBCL) accounts for 35% of all lymphoid neoplasms [1,2]. DLBCL is an aggressive mature B cell neoplasm involving predominantly the lymph nodes and other lymphoid tissues such as thymus, Waldeyer’s ring, and spleen (nodal DLBCL). Less frequently, it involves non-lymphatic tissues, a clinical condition called primary extra-nodal DLBCL (EN-DLBCL) [3,4], whose common sites, in decreasing order of incidence, are the stomach, intestine, nose and sinuses, testis, skin, thyroid, central nervous system (CNS), breast, bones, salivary glands, ovary and cervix, oral cavity, kidneys, lung, and orbits [5,6,7].

Primary parotid gland lymphoma accounts for approximately 4–5% of all the primary extra-node lymphomas [8] and 1.7–3.0% of all salivary gland neoplasms, and, on these, primary DLBCL is much less common than primary mucosal-associated lymphoid marginal zone lymphoma (MALT-L). Lymphopoiesis in the salivary glands is most likely due to repeated antigenic stimuli, such as infections or chronic diseases (i.e., the Sjogren’s syndrome).

Primary thyroid lymphoma accounts for approximately 5% of all thyroid neoplasms and 3% of the extra-nodal lymphomas [9,10,11,12], DLBCL being the most common histology [13]. The only risk factor associated with this neoplasm is the Hashimoto chronic autoimmune thyroiditis [14,15].

According to the most accredited guidelines (ESMO, AIOM, NCCN) [16,17,18], the diagnosis of lymphoma should be made by experienced hemato-pathologists examining an excisional or incisional surgical biopsy [19]. Like DLBCLs in lymphatic sizes, primary non-voluminous EN-DLBCL (stage IE according to Ann Arbor classification), both in the parotid gland and in thyroid, should be treated with three to four courses of R-CHOP (rituximab, cyclophosphamide, doxorubicin hydrochloride, vincristine sulfate, and prednisone) chemotherapy [20,21] followed by radiotherapy of the involved site (ISRT). Six cycles of R-CHOP are considered a good choice if the disease involves a site that it is preferable not to irradiate to avoid complications [22,23,24]. Surgery alone is not enough to treat DLBCL.

According to International Workshop Response Criteria (IWRC), complete remission (CR) of DLBCL implies the elimination of the neoplasm from all involved sites and the disappearance of related symptoms [25]. Patients with PET (Positron Emission Tomography) avid lymphoma (Hodgkin Lymphoma, DLBCL) who obtain a negative PET/CT (Computed Tomography) (with a Deauville index of 1–3) at the end of therapy are considered in CR, regardless of the residual lymph node masses on attenuation correction CT acquired during PET/CT [26].

This paper reports two anecdotal cases of primary EN-DLBCL (one in a parotid gland, and one in the thyroid) treated exclusively with surgery resection, given the inability to apply appropriate chemotherapy in a patient and the stubborn rejection of chemotherapy by the other. These patients achieved complete remission, maintained until now after an 18-month follow up. All procedures have been carried out in compliance with the Helsinki declaration and the Italian privacy laws. All patients signed an anonymous informed consent for the use of their data for anonymous clinical investigation and scientific publication.

## 2. Case Report 1

**Figure 1:**
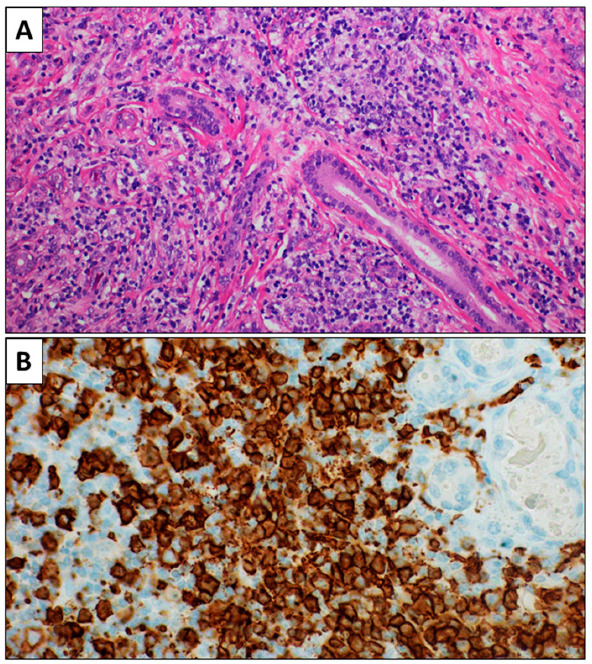
(**A**): Histological examination showing a lymphoid population in a fibrotic contest. A glandular duct is present (H&E, 20×). (**B**): Glandular lobules in the context of the lymphoid population are evident (CD20 immunohistochemistry, 40×).

A 73-year-old Caucasian male with hypertension and chronic kidney disease presented with left cheek swelling in November 2015, which progressively increased over the next 2 months. His medical history was marked by a bladder neoplasm treated in 2008 with total cystectomy and reconstruction, complicated by intestinal adhesions that required ileo-cutaneostomy. An echocardiogram showed lower basal tract akinesia probably due to a previous heart attack; the ejection fraction was 55%. An ultrasound examination (US) and an MRI revealed an increase in the volume of the left parotid within which there were two hypertrophic lymph nodes of about 2 × 2 cm^2^ that had an increase in the absorption of fluorodeoxyglucose (FDG) at a PET/TC. Sjogren’s syndrome was clinically excluded. The patient was in stage IE according to Ann and Arbor staging, with International Prognostic Index (IPI) for Diffuse Large B-cell Lymphoma of 2 (low-intermediate risk group). Routine laboratory tests were normal except for serum creatinine (2.78 mg/dL). Serum markers of HCV, HBV, and HIV infections were performed and found negative. The excision of the left parotid was performed at the Maxillofacial Surgery Unit of our University at the end of January 2016. The histological examination revealed a diffuse lymphoid population composed of large cells, filling the glandular parenchyma in a fibrotic context (Figure 1). On immunohistochemical examination, large cells showed positivity for CD20, CD10, and MUM1, and negativity for CD3, bcl6, CD5, bcl2, CD30, CD21, and CD23. The proliferative index (Ki67) was about 50%. The diagnosis of non-Hodgkin’s DLBCL lymphoma was made according to WHO criteria of 2017. Based on its immunohistochemical profile, this lymphoma was considered Germinal Center B-cell like (GC-type) according to Hans’ algorithm [27]. The pathologists diagnosed non-Hodgkin DLBCL GC-type lymphoma in accordance with the 2016 WHO’s criteria (Figure 1).

**Figure 2:**
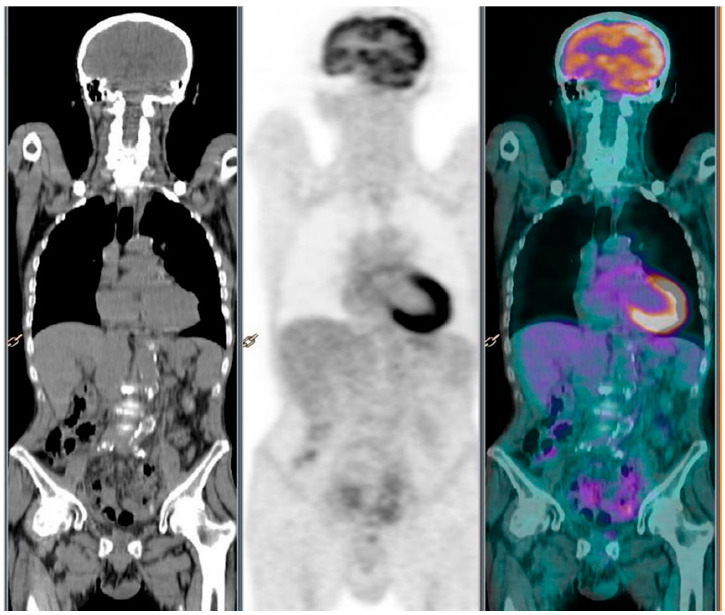
The PET/CT scans performed in December 2017 do not show pathologic masses with high glucose metabolism.

In March 2016, no fluorodeoxyglucose (FDG) uptake was detected in the resection area on a PET/CT scan. In May 2016, the patient deteriorated to a grade 3 ECOG (Eastern Cooperative Oncology Group) performance status, with severe asthenia and electrolyte imbalance, which prevented chemotherapy from starting. This worsening was not due to a reactivation of the lymphoma, as evidenced by the absence of any swollen lymph nodes on physical examination and on a US performed in July 2016. Additionally, there was no fever, night sweats, weight loss, and itching skin. He was treated with intravenous injections of electrolytes, glucose, branched-chain amino acids, B vitamins, and folate every day for 2 weeks. A possible reactivation of the lymphoma was further ruled out by three PET/CT scans performed in September 2016, February 2017, July 2017, and December 2017, all negative for FDG absorption in all possible sites of onset of lymphoma, suggesting a complete remission without chemotherapy or RT (Figure 2). Free from lymphoma since January 2016, the patient suffered a second episode of cardiac ischemia in November 2018 and died of heart failure.

## 3. Case Report 2

**Figure 3:**
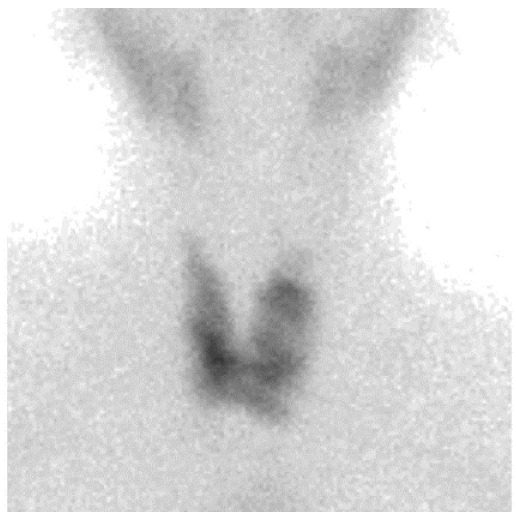
Thyroid scintigraphy with TC-99 74 MBq iv: Glandular parenchyma of the thyroid is not homogeneous, due to the presence of various hypocaptic areas in the context of both lobes, of which the two largest were located one in the right upper lobe and the other in the middle third and at the base of the left lobe.

**Figure 4:**
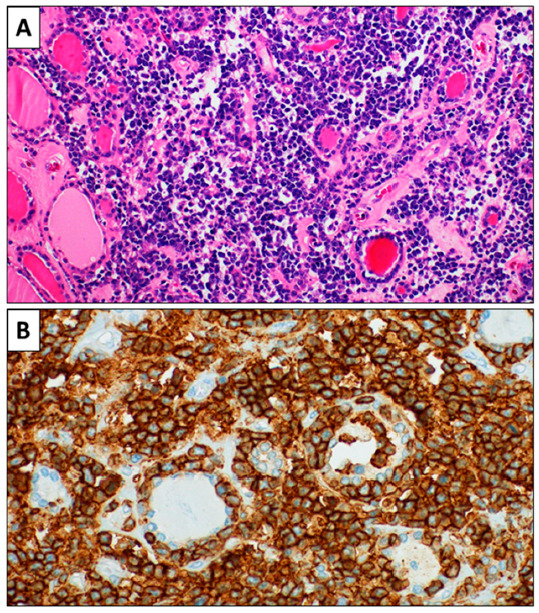
(**A**): Histological examination showing a dense lymphoid population constituted by large cells. Some thyroid follicles are recognizable on the left side (H&E, 20×). (**B**): The lymphoid cells show immunohistochemical expression of CD20. Some CD20-negative thyroid follicles are present in the context. (CD20 immunohistochemistry, 40×).

In November 2017, a 77-year-old Caucasian woman had a strong feeling of weight in her throat. An ultrasound examination reviled an enlarged thyroid gland with some nodules inside, the largest of which had a 14.5 mm in diameter and was vascularized. A fine needle aspiration performed in February 2018 showed no malignant cells. A few days later, a thyroid scan demonstrated some low-absorption areas in both lobes (Figure 3), with the two largest being one in the right upper lobe and the other in the middle third and base of the left lobe. Compression symptoms increased over time and total thyroidectomy was performed at the end of July 2018. Histological examination showed a dense large cells lymphoid population, including some residual thyroid follicles. (Figure 4). On Immunohistochemical examination, the large cells showed positivity for CD20 and MUM1, and negativity for CD3, CD10, bcl6, CD5, bcl2, CD30, CD21, and CD23. The proliferative index (Ki67) was around 50%. The diagnosis of non-Hodgkin DLBCL lymphoma was made, according to the WHO criteria of 2017. Based on the immunohistochemical pro-file, the lymphoma was considered non-Germinal Center B-cell type(non-GC-type) according to the algorithm by Hans. Pathologists diagnosed non-GC-type DLBCL according to the 2016 WHO criteria (Figure 4). On September 2018, a PET/CT scan showed no recurrence of the disease. The patient was in stage IE according to Ann and Arbor staging with IPI 1 (low risk group).

**Figure 5:**
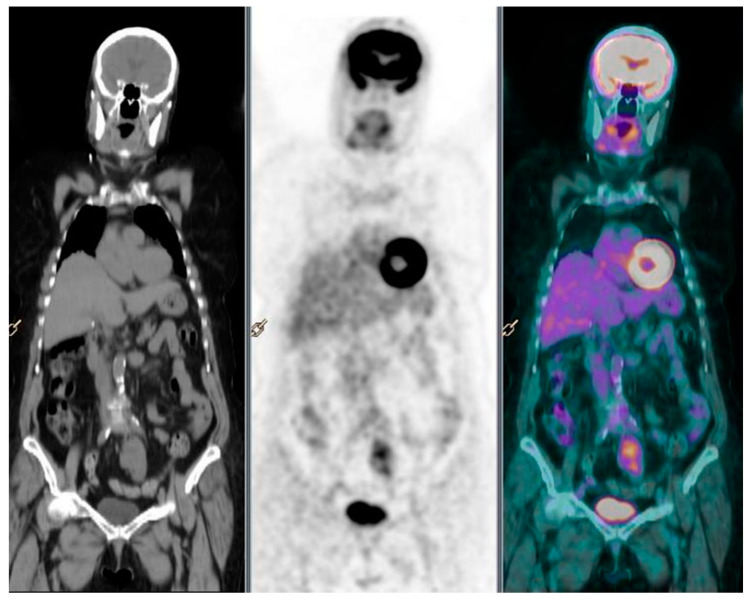
PET/CT scans performed at October 2020: The examination does not show pathologies with a high glucose metabolism.

A blood chemistry test performed on 10 May 2018 showed a sedimentation rate (ESR) of 3 mm, C-reactive protein 4 mg/L, fibrinogen 250 ng/dL, lactate dehydrogenase (LDH) 200 U/L, and albumin 3.6 g/dL. Blood count, triglyceride, cholesterol, and glycemic levels were at the lower limits of normal. An echocardiogram performed a week later, showed a good heart function. The patient was also tested for serum markers of HIV, HBV, and HCV and found positive only for anti-HBc, a sign of previous HBV infection. Lamivudine prophylaxis at a dose of 100 mg/day was initiated 4 weeks prior to the intended initiation of chemotherapy to prevent HBV reactivation [28,29,30,31,32,33,34], and detection of HBsAg, anti-HBs, alanine aminotransferase, and HBV Serum DNA was scheduled at 4-month intervals during chemotherapy. Meanwhile, a second PET/CT scan showed no recurrence of disease. After this favorable result, the patient opposed a stubborn refusal to chemotherapy and the lamivudine prophylaxis was discontinued. In October 2019, she had a PET/CT scan that showed no absorption. A further PET/CT scan performed in October 2020 was still negative (Figure 5). At present, the patient is in good clinical condition and still refuses chemotherapy.

## 4. Discussion

According to the most accredited guidelines (ESMO, NCCN, AIOM), patients suffering from NHL DLBCL with non-bulky disease, stage IE (according to Ann Arbor classification) should be treated with three to four courses of R-CHOP chemotherapy, followed by ISRT, a treatment to be used even for DLBCL localized in extra-lymph node sites (CNS excluded). According to IWRC and ESMO criteria, complete remission (CR) implies the disappearance of all neoplastic lesions and all related symptoms. Patients with avid PET lymphoma (Hodgkin Lymphoma, NHL DLBCL) who show a negative PET (with a Deauville index of 1–3) at the end of therapy should be considered in CR, regardless of residual lymph node masses on CT.

Both patients presented here had DLBCL located exclusively in extra-nodal sites (parotid gland and thyroid) and were treated surgically, and they achieved complete remission without chemotherapy. Both patients, however, were followed up for a relatively short time for DLBCL stage IE, which does not allow to conclude that surgery alone might be sufficient for a complete remission. Accordingly, only a few patients with DLBCL are described in complete remission after surgery alone in literature. It should also be considered that, even if an extra-nodal lymphoma is the only neoplastic lesion detected with accurate diagnostic procedures, the possibility of an undiagnosed systemic involvement may not be excluded, and systemic therapy should be in any case applied [35,36,37,38,39,40].

However, to try to understand the reason for the favorable hematologic outcome in our two patients, some further consideration is useful. Both patients had a low-risk IPI (international prognostic index) and prognostically favorable extra-nodal site involvement [41,42]. Consequently, it is possible that the disease was at the beginning in both cases and that the surgical resection alone was sufficient to obtain complete remission. Another aspect that should not be underestimated is the histological variability of the DLBCL [43,44,45,46,47,48,49,50,51,52,53,54,55,56,57,58,59,60,61,62,63], the classification of which is still under evaluation.

## 5. Conclusions

It may happen that some patients with primary extra-nodal DLBCL heal only with surgical excision, but it must be kept in mind that in world clinical practice, there are much more people who relapse after chemotherapy or, worse, after reaching the complete remission. The two cases presented here and a few others reported in the literature should be considered exceptions to the rule, but these exceptions should not be underestimated. We hope that this study will stimulate other hematologists to report similar cases to increase our knowledge of primary extra-lymph node DLBCL lymphomas with an unusual therapeutic response, a first step towards a multicenter study to identify which of the patients unable to resist chemotherapy and/or ISRT can benefit from surgical excision alone.

## Data Availability

Not applicable.
